# The Erie County Medical Society

**Published:** 1881-01

**Authors:** 


### THE ERIE COUNTY MEDICAL SOCIETY.

This society holds its annual meeting at the Medical College, on Tuesday, January 11. Dr. F. F. Hoyer, the President, will preside. The usual routine business, which possesses but little of professional interest, will probably occupy, as in the past, much of the time devoted to the meeting. We have intimated above that the society, acting for the profession in its corporate capacity, would be profitably employed in devising judicious measures for the enforcement of the medical law. The subject is of sufficient importance to elicit general discussion, and to bring out especially the views and opinions of its older and experienced members. Whatever is done in this important matter should be well considered, in order that the profession may not compromise the dignified position it now maintains towards the public.

